# Zika in the United States: How Are We Preparing?

**DOI:** 10.1289/ehp.124-A157

**Published:** 2016-09-01

**Authors:** Charles W. Schmidt

**Affiliations:** Charles W. Schmidt, MS, an award-winning science writer from Portland, ME, writes for *Scientific American*, *Science*, various *Nature* publications, and many other magazines, research journals, and websites.

In early 2015 clusters of patients started showing up in clinics throughout northeastern Brazil with symptoms of what seemed to be dengue fever: skin rashes, joint pain, and headaches.^1^ ^,^ ^2^ That wasn’t unusual, because mosquito-borne dengue fever has been endemic in Brazil for over 30 years.^3^ ^,^ ^4^ But these cases were uncharacteristically mild. Brazilian scientists were intrigued, so they analyzed blood samples from symptomatic patients and found they were infected not with dengue virus but with a closely related virus called Zika. Brazil’s first case of Zika was confirmed on 15 May 2015, and by the end of the year, cases in that country had shot up to as many as 1.3 million.^5^

**Figure:**
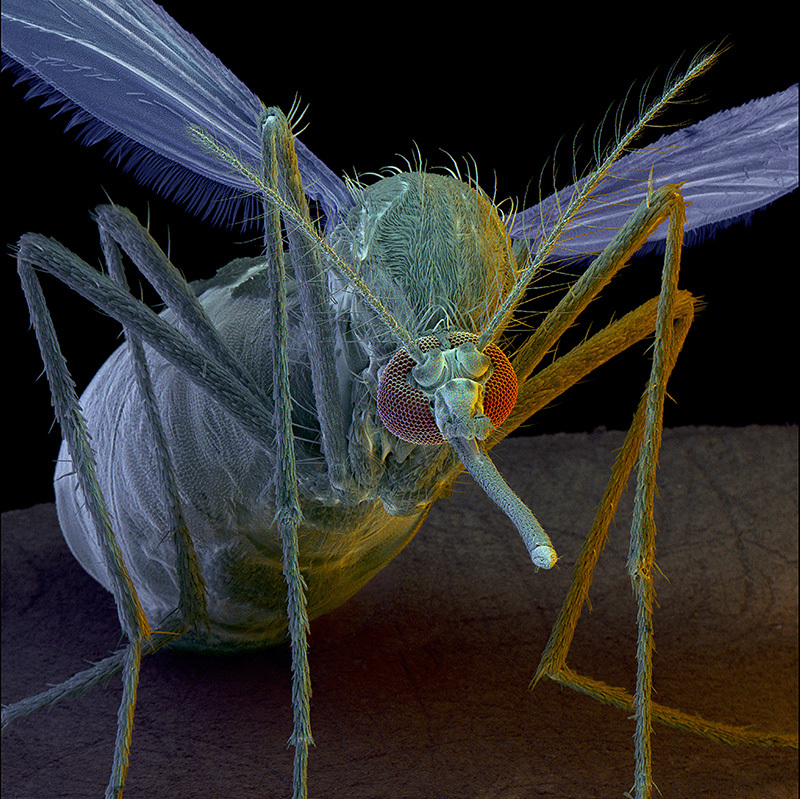
Once considered an inconsequential illness, Zika is now linked with serious health effects including microcephaly and Guillain-Barré syndrome. As the virus makes its way northward, U.S. public health agencies are doing their best to prepare. © David Scharf/Science Source

Public health officials in the United States generally predict far fewer cases here. But the discovery that Zika infection in pregnant women can cause microcephaly, a severe birth defect, has prompted widespread concern and continuous media coverage. According to Anthony Fauci, director of the National Institute of Allergy and Infectious Diseases (NIAID), part of the U.S. National Institutes of Health (NIH), protecting pregnant women from Zika infection is now an overarching priority for local, state, and federal health officials trying to prevent the virus from spreading.

## Emerging Evidence

Zika had been considered an inconsequential illness from which people can easily recover (indeed, only 1 in 5 infected people will exhibit any symptoms whatsoever^6^). That changed with the recent Zika epidemic in Brazil and an earlier one in French Polynesia, which were accompanied by serious and unprecedented complications.

The French Polynesian outbreak sickened an estimated 32,000 people in 2013 and 2014.^7^ In this outbreak, Zika infection was associated with elevated risk of Guillain-Barré syndrome, a neurological condition that produces temporary paralysis.^8^ The association with microcephaly, meanwhile, was detected in Brazil.^9^ Microcephaly occurs when brain development stops prematurely in the womb. Microcephalic children have heads that are smaller than normal, and they are prone to seizures, developmental delays, intellectual disabilities, and other problems that can require lifelong care.^10^

**Figure:**
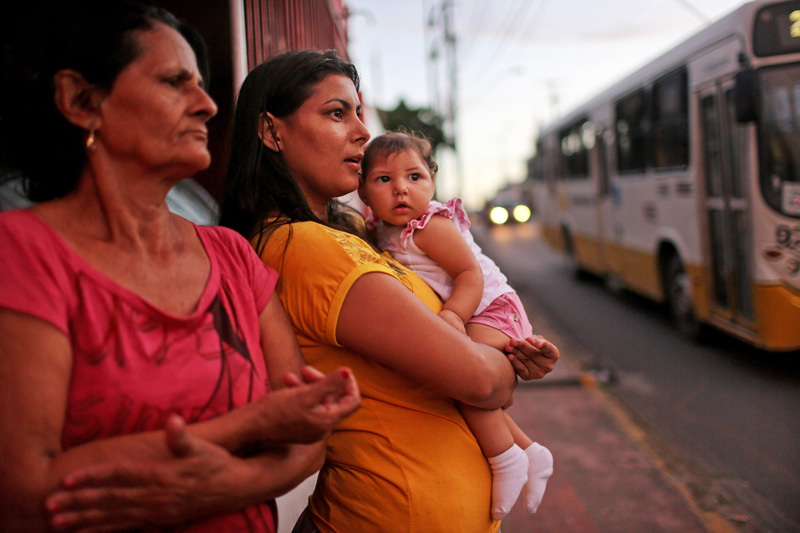
A Brazilian mother in Recife holds her 7-month-old daughter, who was born with microcephaly. Recife has been one of the cities hit hardest by Zika. Public health officials in the United States generally predict far fewer cases here than elsewhere in the Americas. © Mario Tama/Getty Images

In April 2016 the Centers for Disease Control and Prevention (CDC) concluded there is a causal relationship between Zika and microcephaly.^11^ “We almost never use the word ‘causal’ in birth defects epidemiology,” says Margaret Honein, co–team leader of the CDC’s Pregnancy and Birth Defects Team and 2016 Zika Virus Response Team. “It’s unusual that we would have such strong evidence.”

Baseline rates of microcephaly in the general population are low, ranging from 2–12 babies for every 10,000 live births in the United States.^12^ Ernesto Marquez, a medical doctor with joint appointments at the University of Pittsburgh’s Center for Vaccine Research and Brazil’s Oswaldo Cruz Foundation, says that roughly 1,700 cases of confirmed microcephaly were recorded in Brazil as of this summer, the vast majority in northeast regions of the country where the Zika epidemic began. That corresponds to a microcephaly risk of approximately 5–10% among Zika-infected pregnant women in northeast Brazil. “But that’s just an educated guess,” Marquez emphasizes, given that the number of mothers who were actually infected with Zika is unknown. By comparison, Brazil recorded 147 cases of microcephaly in 2014, or around 0.5 cases for every 10,000 births, although some experts say that’s an underreported figure since the average baseline rate is about 10 times higher than that seen in other countries.^13^

Just how much the risk of microcephaly increases with Zika infection is still being determined. In Polynesia^14^ about 1% of babies born to Zika-infected women had microcephaly (or 1 out of every 100 births), while a study that used ultrasound to identify babies with microcephaly among Zika-infected women in Brazil^15^ suggested that 30% of babies were affected. “We’re going to learn a lot more about the risk of adverse outcomes as more data become available,” Honein says.

**Figure:**
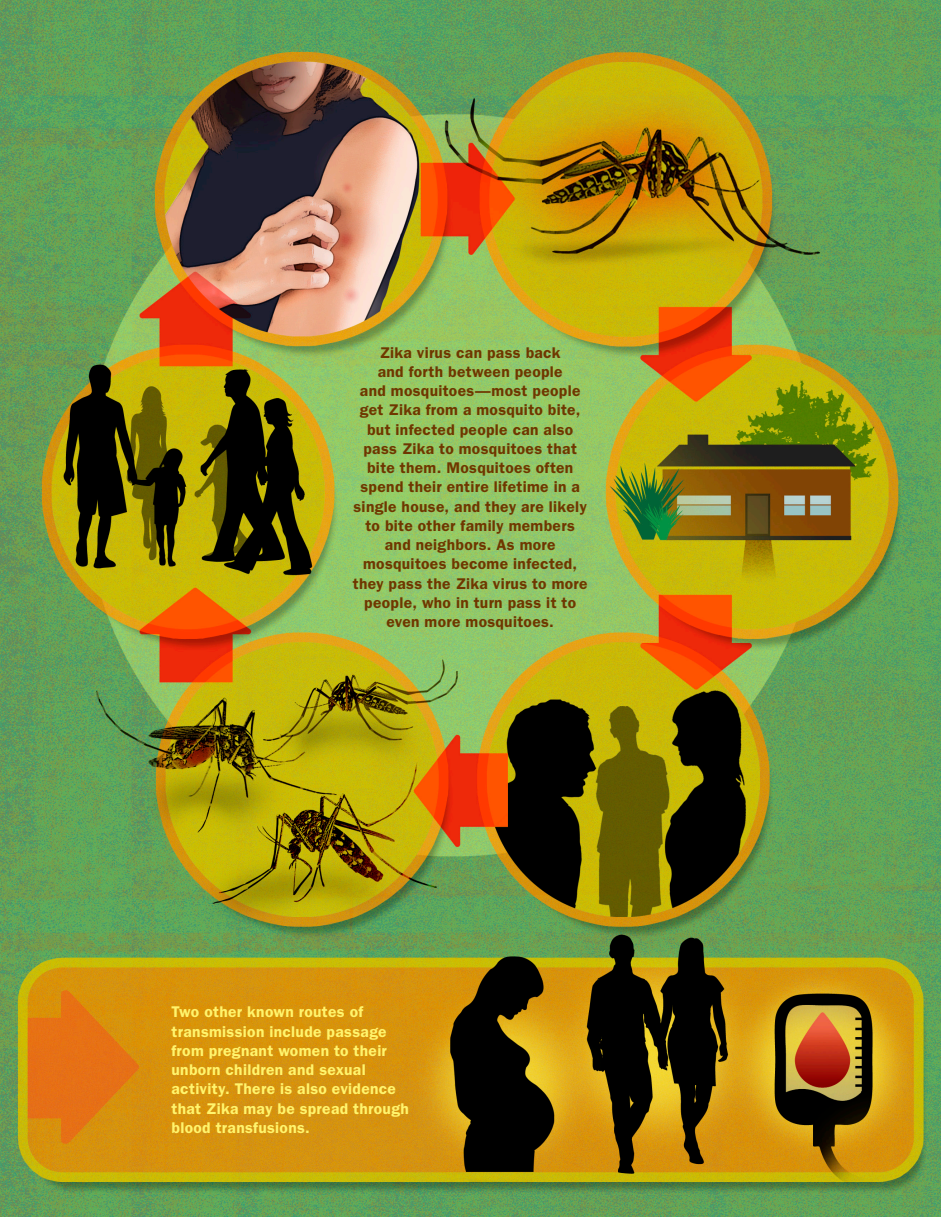
Zika virus can pass back and forth between people and mosquitoes—most people get Zika from a mosquito bite, but infected people can also pass Zika to mosquitoes that bite them. Mosquitoes often spend their entire lifetime in a single house, and they are likely to bite other family members and neighbors. As more mosquitoes become infected, they pass the Zika virus to more people, who in turn pass it to even more mosquitoes. Two other known routes of transmission include passage from pregnant women to their unborn children and sexual activity. There is also evidence that Zika may be spread through blood transfusions. Roy Scott for EHP

Zika only recently became a “notifiable” illness in Brazil, meaning doctors must report cases to the health ministry. As of early 2015, most Zika infections were still being reported as dengue fever, and moreover, “since Zika symptoms are mild, most people didn’t feel like they needed to go the doctor,” Marquez says. “So the total number of infections could in reality be much higher than what’s been recorded so far.”

The number of confirmed Zika infections in Brazil is up to 90% higher among women than men in the 15–65 age group. However, experts caution that since women are more likely than men to visit a doctor, they’re also more likely to be tested.^16^ Now that Zika has become notorious in Brazil, Marquez says it’s likely gone from being underreported to being overreported.

Yet another problem complicating risk estimates is that during the epidemic’s early days clinicians lacked the training needed to correctly diagnose microcephaly. For instance, Marquez points out that cranial circumference shouldn’t be measured until several hours after delivery, since the baby’s head undergoes a normal and reversible compression while passing through the birth canal.

Zika infections in Latin America and the Caribbean (including the U.S. territory of Puerto Rico) have for the most part been locally acquired. In contrast, nearly all of the 2,260 cases recorded in the continental United States as of 17 August 2016 were associated with travel to affected countries or sex with an infected person,^17^ although in July Florida health officials began reporting locally acquired cases in Miami.^18^

**Figure:**
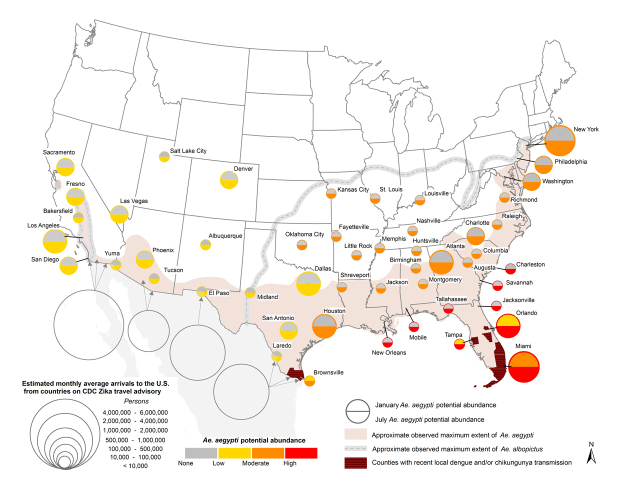
Scientists are investigating other species as potential carriers, including *Ae. albopictus* and *Culex quinquefasciatus* (whose range, not shown, extends across the southern United States). Although these species have been shown to carry Zika in the wild, they are also less likely than *Ae. aegypti* to bite people. Map: Monaghan et al. (2016)^18^

**Figure:**
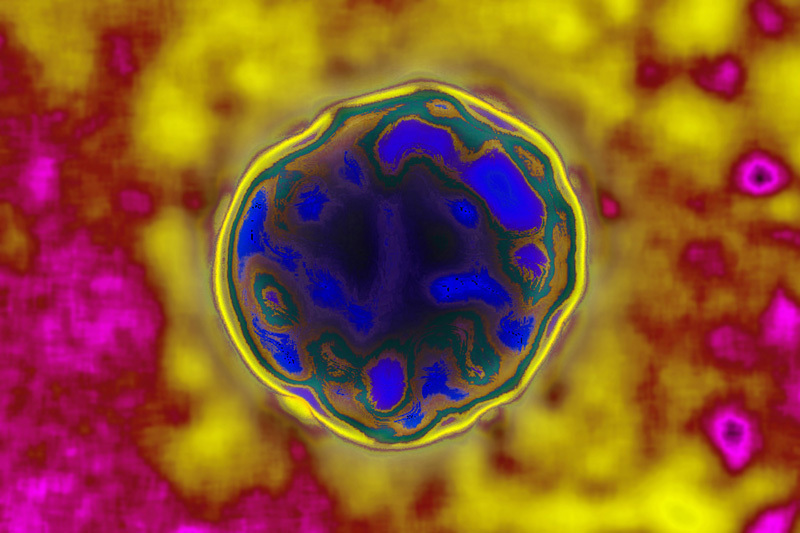
Not all mosquitoes can become infected with Zika virus—it requires specific cell membrane receptors that only some species have. Aedes aegypti is currently considered the major vector of Zika virus in the Americas. © James Cavallini/Science Source

Researchers interviewed for this article don’t anticipate a widespread Zika epidemic in the continental United States, chiefly because the principal Zika vector, the *Aedes aegypti* mosquito, has a limited seasonal range in this country.^19^ Models that map where *Ae. aegypti* might be found in the United States support that view.^19^ Moreover, U.S. households tend to be better protected by doors and window screens than dwellings in countries farther south. Citing prior experience with dengue and chikungunya, two other viral diseases transmitted by *Ae. aegypti*, Fauci says Zika caseloads in the United States as of August 2016 are consistent with his earlier predictions. “We expected some local transmission of individual cases or clusters of cases in the southern Gulf States, and that’s exactly what’s happening in Florida now,” Fauci says.

But those working to constrain Zika’s spread are also dealing with a virus about which little is known, emphasizes Brian Foy, an associate professor of microbiology, immunology, and pathology at Colorado State University. He says there is an urgent need to understand the broad mechanisms by which Zika infections produce microcephaly and, potentially, Guillain-Barré syndrome. “That could help us design more competent countermeasures,” Foy says, “since right now all we can tell people is to avoid getting bitten by mosquitoes or to avoid having sex with a potentially infected person, which isn’t terribly useful.”

**Figure:**
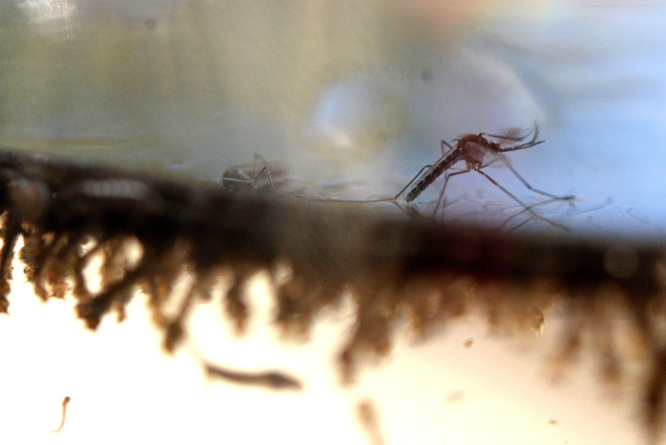
A male mosquito emerges from its watery nursery and waits for its body to dry and harden before flying away. The males live for only about a week. © Marvin Recinos/AFP/Getty Images

**Figure:**
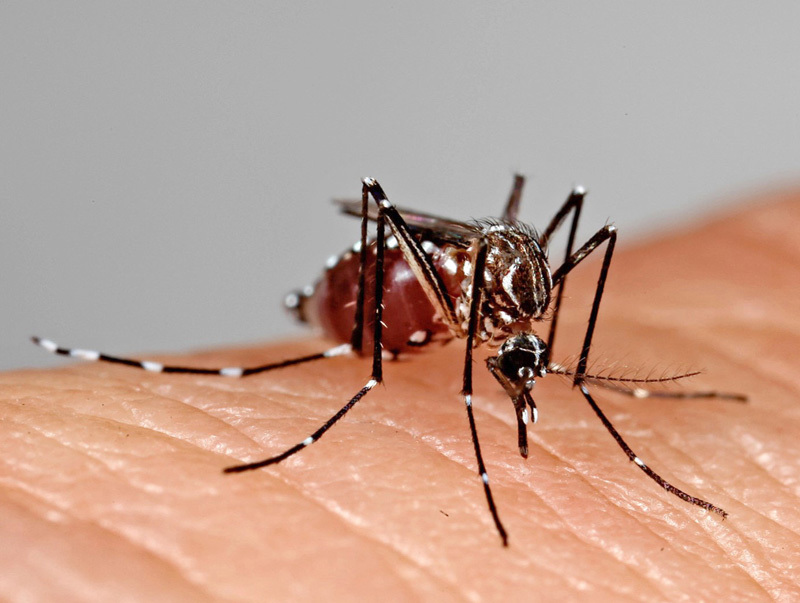
Only female mosquitoes bite; they need the iron and other nutrients in blood to produce eggs. Both sexes meet their own nutritional needs with flower nectar. Photo courtesy of Ed Freytag, New Orleans Mosquito, Termite and Rodent Control Board

## How It Spreads

Zika virus was first discovered in 1947, when it was isolated from a rhesus monkey in Uganda.^20^ It is one of 53 species in the genus *Flavivirus*, which also includes the viruses responsible for dengue fever, West Nile fever, and yellow fever. Until 2007, when the first outbreak of Zika occurred on the Pacific island of Yap, just a handful of cases had been reported, all of them in Africa and Asia.^1^ ^,^ ^21^ ^,^ ^22^

In July 2016 scientists reported that Zika had been detected in wild *Ae. aegypti* mosquitoes in Chiapas State, Mexico, where an outbreak of the illness had begun some months earlier. According to the authors, the results for the first time implicate *Ae. aegypti* as a principal vector of Zika in North America.^23^

*Ae. aegypti*, is an invasive domestic mosquito with African origins that ranges throughout tropical and subtropical areas worldwide. It is thought to have arrived in the Western Hemisphere on ships hundreds of years ago. Until it was partially displaced in the United States by more aggressive competitors, *Ae. aegypti* produced historical outbreaks of yellow fever, an illness that is now preventable with vaccines.^24^ Frank Welch, medical director at the Louisiana Department of Health, describes *Ae. aegypti* as “a pesky little mosquito that doesn’t travel very far and likes to be around people.” It thrives in impoverished neighborhoods with poor sanitation, flying through open doors and windows and breeding near stagnant pools of water in old tires and other rubbish.^19^

A mosquito can transmit Zika only if the virus is able to infect its gut and then spread to its salivary glands—and that’s a species-specific phenomenon that depends on whether mosquitoes have the right cell receptors, according to Thomas Scott, a professor in the Department of Entomology and Nematology at the University of California, Davis. Scientists do suspect that other mosquitoes could potentially transmit Zika to humans.

**Figure:**
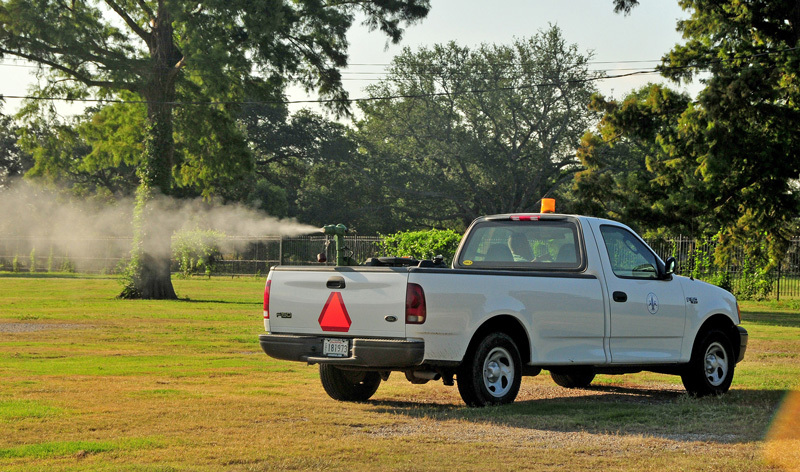
Cities such as New Orleans have adopted a multipronged approach to mosquito control. One component is ground and aerial spraying of pesticides including naled and malathion. Photo courtesy of Ed Freytag, New Orleans Mosquito, Termite and Rodent Control Board

**Figure:**
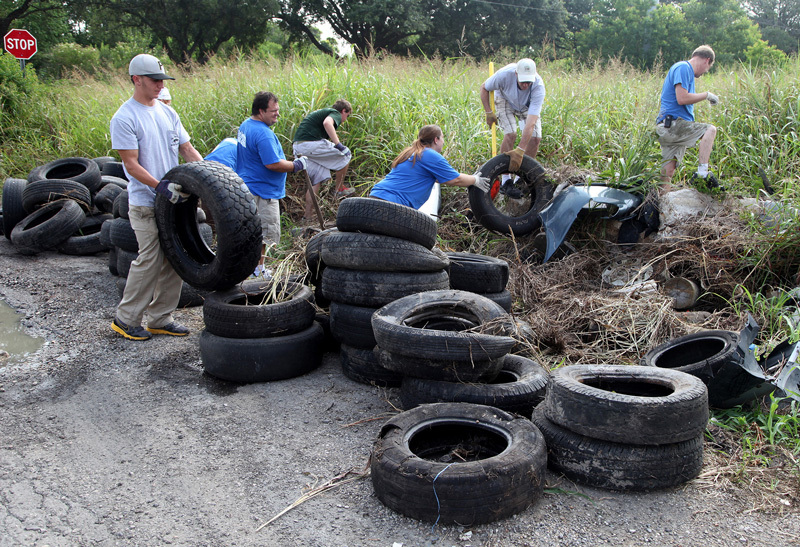
The main focus is on reducing sites where mosquitoes can breed. Here personnel and summer interns from the New Orleans Mosquito, Termite and Rodent Control Board collect discarded tires in the Lower 9th Ward as part of a community engagement project. Photo courtesy of Ed Freytag, New Orleans Mosquito, Termite and Rodent Control Board

**Figure:**
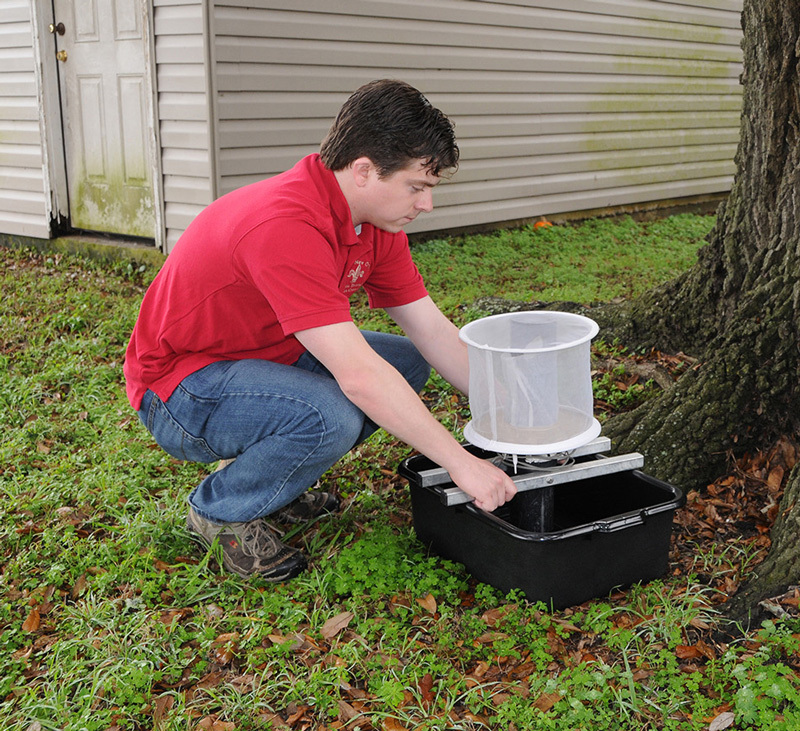
City staff also track the presence of various viruses in local mosquito populations. This entomologist is trapping Culex mosquitoes, which transmit West Nile virus. Photo courtesy of Ed Freytag, New Orleans Mosquito, Termite and Rodent Control Board

One candidate, Scott says, is *Ae. albopictus,* better known as the Asian tiger mosquito, which transmits dengue and chikungunya.^25^ Another is *Culex quinquefasciatus,* the southern house mosquito, a vector for West Nile virus and the viruses that cause Japanese and St. Louis encephalitis.^26^ Laboratory studies show that Zika can infect both these types of mosquitoes, suggesting human transmission from their bite is theoretically possible.^27^

Researchers earlier this year detected Zika in a small number of *Ae. albopictus* mosquitoes captured in central Mexico,^28^ and in July Brazilian researchers identified the virus in wild *Cx. quinquefasciatus* mosquitoes.^29^ Both species are cold tolerant, with ranges extending well into New England and even Canada. But as potential Zika vectors, they share a major shortcoming in that they feed on a variety of birds and animals in addition to people.

Scott explains that threats posed by any mosquito depend heavily on its human biting rate, or the percentage of its diet that comes from the blood of humans instead of other species. *Ae. albopictus* and *Culex*, he says, have low human biting rates, “and that significantly reduces the chances that they will become infected with Zika and then transmit the virus to human populations.” By contrast, *Ae. aegypti*’s human biting rate is 100%, which increases the chances that it can pick up the pathogen from one person and pass it on to someone else.

The bigger concern is that Zika—like no other mosquito-borne virus has been known to do so far—can spread by sexual transmission.^30^ “We’re worried especially about silent reservoirs of asymptomatic people who are also sexually active,” says Steven Presley, a professor in the Institute of Environmental and Human Health at Texas Tech University. “They might infect multiple people before they themselves know they have Zika.”

The first report of sexual transmission of Zika was published in 2011,^31^ and it described the case of an asymptomatic man who infected his wife upon returning from southeastern Senegal. While there, he had been bitten repeatedly by what Foy says were likely *Ae. vittatus* or some other common African tropical *Aedes* species found in rural villages. Sexual relations took place before the husband developed Zika symptoms 6–9 days after his return. Zika transmission through sexual contact has since been reported in the United States, France, Italy, Argentina, Chile, Peru, Portugal, New Zealand, Canada, and Germany.^32^ More recently, scientists confirmed that Zika can be spread by vaginal, anal, and oral sex, and possibly by sharing sex toys, although there is currently no evidence that it can be transmitted by kissing.^30^ ^,^ ^33^

When asked how much sexual transmission might contribute to a potential Zika outbreak in this country, Foy replies, “That’s the forty-million-dollar question.” Scientists simply don’t have enough information to generate a reliable estimate, he says. Once thought to pass only from men to women during sex, there is now evidence that Zika passes from women to men and from men to men, even in the absence of symptoms.^34^ The incubation period for Zika is thought to be a few days.^35^ The virus clears from blood in about a week, but it persists in semen for at least 2 months after disease symptoms resolve.^1^

A sexually transmitted foothold for Zika in the United States, Foy says, could make the virus harder to eradicate, especially from isolated subgroups. “One could imagine Zika getting loose and persisting in populations of sex workers, for instance,” he says.

## Controlling the Disease

In June 2016 the World Health Organization (WHO) issued guidelines urging people who live in the 46 Latin American and Caribbean countries where Zika transmission is expected or ongoing to delay becoming pregnant in order to avoid birth defects.^32^ In the United States, however, where local transmission is a lesser problem, health officials are urging less dramatic precautions to ward off potential outbreaks. In January 2016 the CDC issued a Level 2 travel alert,^36^ which urges people traveling to high-risk countries and regions to take precautions to avoid mosquito bites (A Level 1 travel alert, on the other hand, would simply advise people to consider delaying their travel.)

The CDC has no plans to issue a Level 3 alert, that is, generally recommending against nonessential travel to these areas, according to spokesman Benjamin Haynes. But the CDC does state that pregnant women should not travel to such areas, and those who must travel should first confer with their doctors, and then take strict measures to avoid mosquito bites upon arrival.^37^ Similarly, the CDC cautions people who have recently returned from high-risk areas to use barrier contraceptives or avoid sex if pregnancy is at issue—for men who exhibit symptoms, the duration of this recommendation is 6 months; for others, 8 weeks is recommended.^38^

Vulnerable U.S. Gulf states are now under the spotlight in terms of U.S. cases. In Florida, high-risk areas include Miami–Dade County (especially a square-mile area in the Wynwood neighborhood), the city of Jacksonville, and Key West.^39^ In August 2016 the CDC issued a travel alert recommending that pregnant women avoid the Wynwood neighborhood.^40^ Texas Tech’s Presley says many border towns in southern and western Texas are also at high risk of local Zika transmission.

Both Florida and Texas have been hit in the past by diseases spread by *Aedes* mosquitoes. Florida, for instance, reported 98 cases of locally acquired dengue fever between 2010 and 2016, while Texas reported 24 cases during the same period. But remarkably—and reflecting how impervious the United States tends to be to *Aedes*–transmitted diseases—only 12 cases of locally acquired chikungunya were reported in the United States (all of them in Florida) during an epidemic that produced an estimated 1.2 million infections in 44 Latin American and Caribbean nations from 2013 to 2015.^41^

According to Fauci, the trends seen with dengue and chikungunya predict the likely magnitude of any potential Zika outbreaks in the United States. Similarly, CDC officials predict that up to a quarter of Puerto Rico’s population might become infected with Zika this year, an estimate based on the number of residents there who were infected with chikungunya during the recent pandemic. “Because chikungunya and Zika are similarly spread, this is one of the estimates we use to predict what might happen,” says Haynes. “But no one has a crystal ball.”

Meanwhile, mosquito surveillance and control efforts in the United States are relegated to the states. “Federal agencies provide some guidance and expertise, but the entire infrastructure surrounding vector control is carried out by local municipalities,” says Oscar Alleyne, a senior advisor with the National Association of County and City Health Officials. “It’s very much a ‘fend for yourself’ situation.”

The primary goal of mosquito abatement programs along the Gulf Coast is to contain the spread of *Ae. aegypti*. History shows this is possible. Motivated to quell yellow fever outbreaks, 18 Latin American and Caribbean nations eradicated *Ae. aegypti* during the first half of the twentieth century using DDT and aggressively enforced measures to eliminate breeding sites. Centralized government programs strictly enforced these efforts.^42^ The concentrated push was discontinued in the early 1960s once the mosquitoes were brought under control, but the insects soon reinfested the region after the efforts were abandoned.

According to Jorge Rey, director of the University of Florida’s Medical Entomology Laboratory, the emphasis now is on holding vector populations down to levels so low that the probability of transmitting Zika is sharply reduced. Achieving that goal requires some insecticide use, Rey says, targeted especially at mosquito larvae, but it relies mostly on public education campaigns encouraging homeowners to remove items that collect water outdoors, such as tires, buckets, planters, toys, birdbaths, flowerpot saucers, and trash containers.

*Ae. aegypti* requires a wet–dry cycle for breeding. It lays its eggs on the inside of a container above the waterline, where the eggs can survive for many months; the larvae hatch only when they become covered in water.^43^ Louisiana, Florida, and Hawaii have launched what Welch describes as “once-a-week campaigns” urging residents to tip or remove potential water reservoirs regularly in an attempt to disrupt the mosquito’s 7- to 10-day developmental period. “If you can interrupt the breeding cycle, you’ll drastically reduce the mosquito’s population,” he says.

Health departments throughout the Gulf region are now spreading that message, while encouraging residents to take other precautions. The Mississippi State Department of Public Health, for instance, is urging all travelers returning from areas with ongoing Zika transmission to avoid mosquito bites for 3 weeks.^44^ The state’s epidemiologist, Tom Dobbs, says, “We’re telling these people to stay indoors, wear long pants and sleeves, and avoid yard work.” Similarly, Welch says the Louisiana Department of Health is urging returning travelers to avoid mosquito bites for 2 weeks and to call their providers if they have Zika symptoms.

At the same time, mosquito abatement districts and local governing authorities are devoting whatever resources they can to mosquito control. Janet McAllister, a CDC medical entomologist and advisor on mosquito abatement efforts in Miami, says the agents used for mosquito control alternate between pyrethrin and pyrethroid insecticides and various organophosphates, including naled, malathion, and in vary rare instances chlorpyrifos. (Joseph Conlon, a technical advisor for the American Mosquito Control Association, says DDT is not currently considered for Zika control for three reasons: public perception, potential resistance among *Aedes* mosquitoes, and the availability of viable alternatives.)

As of August 10, applicators in the Wynwood neighborhood of Miami had on two occasions applied naled from the air and pyrethrin and pyrethroids from the ground. McAllister says it’s rare for mosquitoes to develop resistance to these pesticides. She says mosquito abatement strategies are developed with an eye toward mitigating environmental impacts of the pesticides used, such as spraying at times of the day when pollinators are not active.

Pyrethrins and pyrethroids are considered generally harmless to people when used according to directions,^45^ unlike organophosphates, which can be toxic to species other than the targeted insects—including humans. Acute exposures to organophosphates at the wrong time *in utero* can potentially cause problems with cognitive development in childhood.^46^ ^,^ ^47^ ^,^ ^48^ But for women of childbearing age in Zika-infected areas, that risk must be balanced against the risk of a devastating birth defect, says Brenda Eskenazi, director of the Center for Environmental Research and Children’s Health at the University of California, Berkeley.

## Next Steps

In June 2016 the CDC released a draft Zika Interim Response Plan laying out a strategy for working with local authorities should Zika transmission expand from a single locally acquired case to a more widespread local or multi-county outbreak.^49^ Among many other measures, the plan calls for—in the case of a single transmitted case—destroying mosquitoes within 150 yards of the infected person’s home (medical privacy laws prohibit publishing the address of an infected individual), expanding to a mile surrounding any clusters that might emerge.

The plan also details how the CDC’s Laboratory Response Network (LRN) is being deployed against Zika. According to Presley, who directs an LRN facility at Texas Tech University, those laboratories are authorized to screen biological samples submitted by clinics around the country. Clinics submit samples if the screened individual exhibits Zika symptoms and meets epidemiological criteria, such as travel to a Zika outbreak area or sex with a Zika-infected person. However, the polymerase chain reaction testing used by the LRN to detect Zika only works when patients are viremic, meaning the virus is circulating in their blood stream, a period that generally lasts no more than 10 days. If the viremic period ends before PCR is conducted, the result will be negative even if the patient is sick with Zika.

Labs can also test for Zika antibodies in blood during the post-viremic period. But according to Welch, those assays cross-react with other flaviviruses, such as dengue, and that makes them susceptible to false-positive results.

Cross-reactive antibodies may also cause problems in infected individuals. According to UC Davis’s Scott, it’s possible that dengue antibodies circulating in people who were previously sick with that illness could react with the Zika virus and amplify its effects.^50^ This phenomenon is called antibody-dependent enhancement, and it has been studied mostly in the setting of dengue, which is actually an umbrella term for four closely related virus serotypes. Antibodies against one serotype can react to viral particles of another and produce a potentially fatal hemorrhagic fever.^51^

Foy shares Scott’s concerns about antibody-dependent enhancement. “It’s crucial that we test whether prior immunity to other flaviviruses can change Zika outcomes,” he says.

For now, the fight to control the spread of the disease continues, and the severe rains that ravaged Louisiana in August^52^ illustrate how public health agencies must grapple with factors beyond their control. Welch says *Aedes* species prefer fresh water—not floodwater, which has a lot of organic debris in it. Some areas of Louisiana received rain but no flooding, and it is these areas, with their standing water, where the risk of *Aedes aegypti* is increased. That said, he adds that some species do breed in floodwater, including those that carry West Nile virus.

Zika’s spread in the most vulnerable countries remains an open question. According to Scott, viral illnesses like Zika can flame out quickly with what’s known as herd immunity: as more people become infected with and then immune to the virus, the pool of susceptible people drops until the epidemic can no longer sustain itself. Indeed, evidence now shows that Zika infections in Brazil may have already reached their peak.^53^

Alternatively, a vaccine could have the same effect while protecting untold numbers of children. “We’ve made successful vaccines against flaviviruses before,” says Fauci, who expects that Phase I clinical trials for Zika vaccine candidates could begin in late 2016. Fauci says he expects a vaccine could be clinically available by 2018. “We don’t know what’s going to happen with this virus—whether it will explode and go to a low level or stay endemic for a while,” he says. “We have to watch and wait and err on the side of being conservative.”
