# Erratum: “Zika in the United States: How Are We Preparing?”

**DOI:** 10.1289/EHP1096

**Published:** 2016-11-01

**Authors:** Charles W. Schmidt

Environ Health Perspect 124(9):A157–A165 (2016), http://dx.doi.org/10.1289/ehp.124-A157

In the following sentence, the 147 cases of microcephaly reported in Brazil in 2014 were incorrectly characterized as being about 10 times higher than the average baseline rate seen in other countries:

“Brazil recorded 147 cases of microcephaly in 2014, or around 0.5 cases for every 10,000 births, although some experts say that’s an underreported figure since the average baseline rate is about 10 times higher than that seen in other countries.”

The correct information should have read “…since the average baseline rate seen in other countries is about 10 times higher than that.”

*EHP* regrets the error.

